# Psychoeducation Programs to Reduce Preoperative Anxiety in Adults: A Scoping Review

**DOI:** 10.3390/ijerph20010327

**Published:** 2022-12-25

**Authors:** Palmira Oliveira, Catarina Porfírio, Regina Pires, Rosa Silva, José Carlos Carvalho, Tiago Costa, Carlos Sequeira

**Affiliations:** 1 Centro de Investigação em Tecnologias e Serviços de Saúde, Escola Superior de Enfermagem do Porto, 4200-072 Porto, Portugal; 2 Hospital Pedro Hispano, 4464-513 Matosinhos, Portugal; 3 Centro Hospitalar de Vila Nova de Gaia, Centro de Investigação em Tecnologias e Serviços de Saúde, 4434-502 Vila Nova de Gaia, Portugal

**Keywords:** adult, nursing, psychoeducation, preoperative anxiety, scoping review

## Abstract

Background: Surgical procedure is a critical event that causes anxiety for patients. One of the possible intervention strategies to reduce anxiety in the preoperative period is psychoeducation. Methods: A scoping review was conducted according to the JBI methodology and PRISMA-ScR to map knowledge about psychoeducation programs to reduce preoperative anxiety in adults. The data were extracted by the researchers, according to the objective of the study. Finally, the data synthesis was presented in narrative format and tables. Results: four studies were included in the review with different characteristics of psychoeducation programs. The approach of these programs consisted of teaching about anxiety, instruction and training in anxiety control techniques. The contents referred to included the surgical process and intervention techniques to reduce anxiety. Program sessions lasted from 45 to 150 min, with a frequency of 1 to 6. The assessment instrument used was the State-Trait Anxiety Inventory. The dynamisers were nurses, psychotherapists and clinical psychologists. Conclusions: Psychoeducation programs can be useful and effective in reducing anxiety. More studies are needed to confirm these results.

## 1. Introduction

According to the American Psychiatric Association [1], anxiety is an emotion that arises when a person anticipates a future threat. In accordance with the International Classification for Nursing Practice, anxiety is a type of emotion with specific characteristics, such as: feelings of threat, danger or anguish with unknown cause, accompanied by panic, decreased self-confidence, increased muscle tension and heart rate, paleness, increased perspiration, palm sweating, dilated pupils and a shaky voice [2]. Anxiety is a person’s habitual response to a threatening situation and, to a certain extent, it is desirable in the sense that it serves to the person’s response capacity [3].

Surgery causes anxiety in many patients because they will be undergoing an unknown experience (for example, anesthesia), are away from home, and can suffer pain, discomfort, and complications [4]. Thus, anxiety is common to most individuals during the preoperative period [5,6,7], regardless of the type of surgery [8], considering that surgical patients experience a higher level of anxiety on the day of the surgery itself [9].

High levels of anxiety can delay the healing of surgical wounds, hinder recovery and negatively affect the quality of life of patients [10] and extreme levels of anxiety can lead to some surgeries being cancelled [11]. Thus, the implementing anxiety assessment scales upon service admission would allow patients with high levels of anxiety to be detected and measures to mitigate them from developing [7]. On the other hand, the Directorate-General of Health reports that, in 2015 anxiety disorders were the sixth most common cause of health loss, 3.4% of years of life adjusted for disability, with approximately 3.6% of the overall population experiencing anxiety disorders [12].

The provision of information in advance, before surgery, allows patients to plan, to consider alternatives for postoperative care, and to identify and ask important questions for their needs, leading to a decrease in their anxiety levels [13]. Therefore, nurses are encouraged to develop models to provide information in the preoperative period in order to reduce anxiety [14].

Preoperative education of anxiety management strategies can lead to a reduction in preoperative and postoperative anxiety as well as postoperative complications [15]. In this way, psychoeducation is a specific form of education that makes it possible to learn facts related to mental illnesses and strategies to deal with them [16]. According to Portuguese regulation No. 515/2018 of the Diário da República [17], specialist nurses of mental and psychiatric health have knowledge and skills in therapy in order to mobilize psychoeducational competences. Specialist nurses of mental and psychiatric health assist the patient throughout their life cycle in optimizing their mental health [17]. Teaching adults to have a unique background of life experience encompassing the disease and understanding the adult learning process is fundamental to develop the education plan carried out by nurses [18].

Given the above, a scoping review (ScR) is an ideal way to assess the current state of knowledge in this field.

## 2. Materials and Methods

A preliminary search was conducted on MEDLINE, PROSPERO, on the Scoping Review Protocol, the Cochrane Database of Systematic Reviews and the JBI Evidence Synthesis, and no ongoing ScR or ScR protocols were identified, reinforcing the need to map the knowledge about psychoeducation programs to reduce pre-surgical anxiety in adults.

We chose to carry out an ScR because we were looking for deep and comprehensive information about psychoeducation, regardless of the study designs (qualitative or quantitative) [19]. ScR is a research method that consists of a kind of synthesis of knowledge following a systematic approach to mapping the evidence on a given topic, and it identifies the main concepts, theories, sources of information and knowledge gaps [20]. With this methodology, we intend to map the knowledge about which psychoeducational programs exist to reduce preoperative anxiety in adults and what are their characteristics.

The present ScR was based on the methodology recommended by JBI [21], and was registered in the Open Science Framework, under the doi: https://doi.org/10.17605/OSF.IO/36KHS (accessed on 29 November 2022). The orientation of the preparation of the ScR was based on the Preferred Reporting Items for Systematic reviews and Meta Analyzes extension for Scoping Reviews (PRISMA-ScR) Checklist [20].

### 2.1. Search for Evidence

The characteristics of evidence sources for the review were defined as eligibility criteria. Using the PCC strategy (Participants, Concept, Context strategy) [20,21], the review considered studies that in terms of Concept described psychoeducation programs developed in the preoperative period, to reduce anxiety; in regarding to the Context, in any type of scheduled surgery, implemented in any setting (hospital, outpatient clinic); and as Participants targeting adults (over 18 years old and under 65 years old). Primary and secondary studies, qualitative and quantitative, published and unpublished, without time constraints, were included. All studies in Portuguese, English and Spanish were searched, since they were the languages of the ScR reviewers’ domain, in order to promote a good quality of evidence selection and data extraction. The exclusion criteria were case reports, opinion articles, text complete unavailable and duplicate reports.

The search strategy was carried out in three phases, between January and February 2020. (I) Initial search in MEDLINE^®^ with Full Text and complete CINAHL database (access via EBSCOhost Web), with the defined Boolean phrase and analysis of the text words in the title and abstracts of the index terms used to describe the article. (II) A search was carried out with all the keywords and indexed terms identified, in the databases and repositories included in the review. (III) Analysis of the bibliographic references of all articles included in the primary search was carried out to identify additional relevant studies.

The databases included in the review were: MEDLINE^®^ with Full Text, CINAHL complete, Psychology and Behavioral Sciences Collection and PsychInfo (access via EBSCOhost Web); Web of Science Core Collection and SciELO Citation Index (access via Web of Science); Scopus; Cochrane library; JBI Evidence Synthesis and the repositories: Scientific Open Access Scientific Repository of Portugal (RCAAP) and OpenGrey (System for Information on Gray Literature in Europe).

#### 2.1.1. Evidence Selection

After removing duplicate articles, the title, abstract and full text were analysed. The selection of articles was carried out independently by two authors. Whenever the abstract raised doubts about the relevance of a study for the researchers, the full text article was retrieved. Articles without an abstract were selected for the relevance analysis of the full text. If disagreement between the researchers was observed, report’s suitability was resolved by a third researcher and by consensus. Finally, additional studies from the reference lists were identified.

#### 2.1.2. Data Extraction

The data were extracted by two independent reviewers, using a tool developed by the researchers, in accordance with the objective of this review. The instrument was created based on the model instrument of the JBI for extracting details characteristics and results [21].

#### 2.1.3. Data Analysis and Presentation

The data were grouped and organized according to the categories defined a priori. Regarding these studies, the objectives and the type of study stand out. From the data related to the intervention programs, the following is highlighted: the participants in the study, the context of implementation, the content, intervention strategy, duration, frequency, time of implementation, program evaluation and anxiety assessment tool, facilitator and outcomes. The data are presented in a more detailed form with figures, tables and a descriptive narration of the results, as recommended by ScR’s recommendations. The PRISMA-ScR flow chart, illustrate the identification, screening and selection process.

## 3. Results

**Figure 1:**
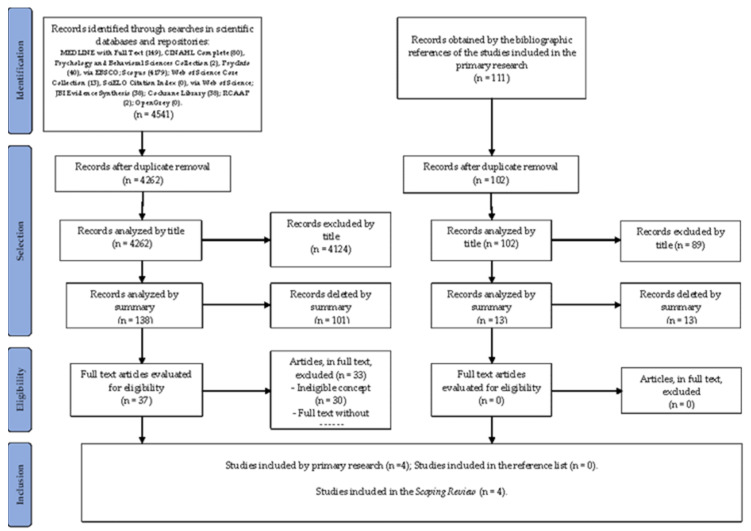
PRISMA- ScR flowchart (adapted) of the study selection process [21].

This section organizes and summarizes the results obtained in the development of the ScR, presented in a visual representation. The search identified 4541 potentially relevant articles, of which 279 were removed for being duplicates; of the remaining 4262 articles, 4124 were excluded by title analysis; of the remaining 138 articles, 101 were excluded after analysis of the abstract; of the remaining 37 articles, 33 were excluded after reading the full text, with 30 articles presenting ineligible population, concept and context and three articles without access to the full text. We finally included four articles in the scope review. In the secondary search of the bibliographic references of the studies included in the primary search, 111 potentially relevant articles were obtained. Nine were eliminated because they were duplicated. Of the remaining 102 articles, 89 were excluded after title analysis; of the remaining 13 articles, all were excluded after analysis by abstract. Zero articles were included in the ScR (Figure 1).

The studies concern primary, published studies, with quantitative methodology, experimental and quasi-experimental, with the aim of evaluating the effectiveness of psychoeducation programs through the State-Trait Anxiety Inventory (STAI) as Anxiety assessment tool. The study by Román et al. [22] was developed in Spain with 50 users undergoing bariatric surgery. The intervention program consisted of six sessions of psychoeducation in group therapy, lasting two and a half hours per session. This program promoted the reduction in preoperative anxiety.

The other studies took place in Iran. The study by Shahmansouri et al. [23] was developed with 60 users who underwent coronary artery bypass grafting. The intervention program consisted of a group psychoeducation session lasting 60 to 90 min. There were no differences in anxiety levels after the intervention. The study by Shakeri et al. [24] was developed with 81 users undergoing orthopedic surgery. The intervention program consisted of a group or individual psychoeducation session. There was a decrease in anxiety levels in the intervention group. The study by Malek et al. [25] took place with 160 users undergoing coronary artery bypass grafting. The intervention program consisted of group therapy psychoeducation sessions, including two sessions lasting 45 to 60 min per session. There was a decrease in anxiety levels in the intervention group.

Concerning the participants and contexts of implementation of psychoeducation programs to reduce preoperative anxiety in adults, they are described in Table 1.

Four studies related to psychoeducation programs to reduce preoperative anxiety in adults were included. These have different characteristics (Table 2).

## 4. Discussion

The intervention strategies included psychoeducation with cognitive behavioral intervention, through relaxation techniques, problem solving, deep breathing and cognitive restructuring, mostly in group therapy. Psychoeducation should include an explanation of the causes of anxiety, physical, cognitive and behavioral symptoms, as well as explaining the treatment, describing assessment methods and intervention techniques used to reduce anxiety [26]. Thus, psychoeducational interventions can be passive or active in reducing anxiety symptoms [27]. The material resources used in the program’s psychoeducation sessions are diverse, ranging from pamphlets, recordings, and images to audios. Many institutions provide information in a standardized pattern in the form of brochures and leaflets [3]. The educational needs of patients undergoing surgical procedures have increased [18]. Health professionals must pay attention to the surgical procedure and know how to personalize care, in order to allow its implementation in order to respond to the patients’ needs [28]. Thus, Gezer and Arslan [29] mention that the selection of training methods, as well as the patients’ needs and characteristics should be taken into account, with individualized care being important.

As for the content covered in the intervention programs of the studies included in this review, precise indications are provided for performing relaxation exercises, information about the surgical process and an exploration and discussion of emotions and anxiety. Preoperative educational texts may vary depending on content, purpose and format [9]. In general, the information provided to patients includes instructions on preoperative care, operative techniques, postoperative expectations, recovery, pain control, postoperative home care [9], as well as relaxation techniques [30]. However, people are different, and the quantity and quality of the information to be provided must be adapted to the patient; it may be important to consider the quantity and type of information to be transmitted, taking into account the characteristics of coping style and locus control, and considering the patients’ family and social context [3].

In terms of frequency of interventions, the interval between sessions was relatively short in the study by Malek et al. [25], taking place on consecutive days, compared to the study by Román et al. [22], in which it was weekly. The frequency of the sessions ranged from one to six sessions, and the duration per session ranged from 45 to 150 min. The learning process, more specifically acquiring knowledge and applying the acquired knowledge, can be conditioned according to the time interval between sessions and the duration of each session.

Regarding the moment of implementing the intervention, there was diversity in the time interval between the moment of implementation and the date of the surgical procedure. In most hospitals, the preparation of the surgical patient and transmission of preoperative information occurs after hospital admission, usually 24 h before surgery, making it difficult to adequately prepare the patient and to assimilate all the information provided [31]. However, carrying out a preoperative nursing consultation allows the nurse to welcome the patient in the therapeutic unit, assess their specific needs and transmit information about preoperative care, the procedure itself and information about discharge and postoperative care [32]. By promoting interaction between the nurse and the patient, as well as the transmission of information, this consultation contributes to reducing the anxiety triggered by the surgery [33], considering that the highest level of anxiety occurs on the day of the surgery [9].

The same anxiety assessment instrument used was in all of the studies (STAI), although the number of items differed. STAI measures anxiety in adults and the study by Shahmansouri et al. [23] and Román et al. [22] reported that the instrument in question had discriminatory validity and good internal consistency. According to Wilson et al. [9], there are authors who advise the use of STAI, due to its ease of use, validity and proven reliability to assess surgical patients’ anxiety.

The dynamisers of the psychoeducation programs to reduce preoperative anxiety in adults were diverse, however, nurses prevailed in relation to the other dynamisers.

Non-pharmacological interventions should be the main functions of nurses’ intervention area [25]. Nurses play an important role in investigating and reducing preoperative anxiety among patients [24]. In particular, specialist nurses of mental and psychiatric health play a pivotal role as a driver of psychoeducation in patients’ mental health.

The results of the programs varied according to the strategies and methods adopted. The results of the study by Shakeri et al. [24] revealed that the planned training intervention reduces preoperative anxiety in surgical patients and the study by Morrell [13] validated the results of the study by Shakeri et al. [24].

As for the participants in the studies, they varied according to the type of surgery. For Renouf et al. [34], preoperative anxiety exists in all surgical patients, regardless of diagnosis. Even in minor surgeries, high levels of anxiety are evident and can affect postoperative results [8].

Concerning the context of implementing intervention programs, they were conducted in an external consultation or during hospitalization. However, in recent years, the number of days of hospitalization has been decreasing with admission generally on the day or on the eve of surgery, so that the period of hospitalization before surgical procedures is short, at 24 h [35].

Despite the results and merit of this review, there are some important limitations, mainly related to the research. On the one hand, only articles in Portuguese, English or Spanish were searched, excluding potentially relevant studies that were published in other languages.

The scarcity of studies found in this ScR also suggests the need to develop more research and publicity about psychoeducation programs that promote the reduction in anxiety in this study context. On the other hand, the results provide a basis for the construction of a psychoeducation program, for its implementation and the evaluation of the impact produced by reducing anxiety levels in adults in the preoperative period through the investigation and execution of a randomized clinical trial.

## 5. Conclusions

This research found four documents about psychoeducation programs to reduce preoperative anxiety. They report studies with different characteristics, carried out in a hospital context. Three present results demonstrate a decrease in anxiety levels in the intervention group and one does not show differences in anxiety levels in the intervention group. Despite the limited number of studies reviewed, the results obtained constitute a contribution of relevant scientific evidence for the construction and development of this type of nursing intervention that promotes health gains. However, more studies are needed to confirm these results.

Regarding the implications for clinical practice, this study can serve as a basis for reflection on the need for specialist nurses in mental and psychiatric health to put their professional skills into action by helping the patient to live their transition processes associated with surgery as healthily as possible, facilitating their adaptive responses and promoting their quality of life.

## Figures and Tables

**Table 1:** Participants and contexts of implementation of intervention programs.

	Román et al. (2012) [22]	Shahmansouri et al. (2013) [23]	Shakeri et al. (2015) [24]	Malek et al. (2018) [25]
Participants	Sample of 50 users; with a mean age of 42.6 (SD ± 9.6) years; undergoing bariatric surgery	Sample of 60 users; with a mean of 59.2 (SD ± 8.7) years in the intervention group and 55.9 (SD ± 10.0) years in the control group; undergoing myocardial revascularization surgery	Sample of 81 users; aged between 18 and 65 years; undergoing orthopedic surgery	Sample of 160 users; with a mean age of 64.79 (SD ± 6.25) years in the intervention group and 64.25 (SD ± 7.75) years in the control group; undergoing myocardial revascularization surgery
Implementation context	Hospital outpatient consultation, in person	Hospital admission, in person	Hospital admission, in person and not in person	Hospital admission, in person

**Table 2:** Characteristics of intervention programs.

	Román et al. (2012) [22]	Shahmansouri et al. (2013) [23]	Shakeri et al. (2015) [24]	Malek et al. (2018) [25]
Intervention strategies	Psychoeducation with relaxation training, cognitive restructuring and problem solving technique; education about the surgical process; in a group of five patients	Psychoeducation with relaxation training, in a group of five to six patients	Psychoeducation with training in problem solving technique and education on the surgical procedure, in group or individual	Psychoeducation with training in anxiety management techniques in groups of 10 patients
Contents	Information on performing relaxation exercises; information on the surgical process, surgical technique and aspects related to hospital admission and monitoring	Exploration and discussion about emotion, anxiety, in a psychotherapeutic atmosphere, with training in relaxation techniques	Provision of a simple and understandable pamphlet, with “training” content and a brief description of the surgery. Filling in a recording, solving a problem or answering a question	Information about the surgical procedure; encouraging the user to talk about anxiety and its causes and correcting users’ mistakes, and education and training in emotion control techniques such as Benson relaxation, deep breathing, guided imagery, repetition of prayers
Frequency and Duration	Six sessions, lasting two and a half hours per session and with an interval of about a week	One session lasting 60 to 90 min	One session	Two sessions lasting 45 to 60 min each session and with an interval of one day
Time of implementation	About six weeks before the date of surgery	On the morning of the first day of hospitalization	24 h before the date of surgery	Two consecutive days prior to the date of surgery
Program evaluation/ Anxiety assessment tool	Pre- and post-intervention through STAI, Spanish version, composed of 40 items	Pre- and post-intervention through STAI, composed of 20 items	Pre- and post-intervention through STAI composed of 40 items	Pre- and post-intervention through STAI composed of 20 items
Dynamiser	Clinical psychologist	Psychotherapist	Nurse	Nurse
Results	Decrease in anxiety levels in the intervention group	No differences in anxiety levels in the intervention group	Decrease in anxiety levels in the intervention group	Decreased levels of anxiety in the intervention group

## Data Availability

All data generated as part of this study are included in the article.
